# GDA-Pred: Generative AI-Driven Data Augmentation for Improved Prediction of IL-6 and IL-13-Inducing Peptides

**DOI:** 10.3390/ijms27177688

**Published:** 2026-08-27

**Authors:** Hiroyuki Kurata, Hiroto Tsuruta, Soyogu Shigetomi, Md. Harun-Or-Roshid, Kazuhiro Maeda

**Affiliations:** Department of Bioscience and Bioinformatics, Kyushu Institute of Technology, 680-4 Kawazu, Iizuka 820-8502, Fukuoka, Japan

**Keywords:** peptide, data augmentation, generative AI, generative adversarial network, diffusion model, variational autoencoder

## Abstract

Identifying interleukin-6 (IL-6) and interleukin-13 (IL-13)-inducing peptides is important for drug discovery targeting cancer, immune disorders, and infectious diseases. However, experimental screening is costly and time-consuming. Machine learning and deep learning models have been developed that distinguish functional peptides from no-function ones, but their performance is limited by the small number of experimentally validated peptides. In this study, we propose a generative AI-driven data augmentation framework, GDA, and its prediction system, GDA-Pred, to improve the performance of state-of-the-art (SOTA) classifiers under limited data. GDA generates peptide sequences using three generative models: generative adversarial networks, diffusion models, and variational autoencoders. The framework is controlled by four hyperparameters: generative model type, sequence identity cutoff, probability threshold, and augmentation ratio. Because optimizing these hyperparameters is difficult with small datasets, we used anti-inflammatory peptide (AIP) data as a proof-of-concept to identify an effective reference hyperparameter setting. We evaluated GDA using stratified 5-fold cross-validation with cluster-based partitioning and a hold-out benchmark test. The GDA with the AIP-derived reference hyperparameter setting was then applied to SOTA classifiers to identify IL-6 and IL-13-inducing peptides as a case study. GDA-Pred consistently improved prediction performance for both cytokine-inducing peptide datasets, demonstrating the potential of generative AI to overcome data scarcity in peptide prediction.

## 1. Introduction

Functional peptides, defined as short amino acid sequences with specific biological activities, have emerged as a promising class of therapeutic agents in modern drug discovery [1,2,3]. Unlike small-molecule drugs, which often exhibit limited specificity and off-target effects, peptides can closely mimic endogenous ligands and protein–protein interaction motifs, enabling highly selective binding to cellular receptors, enzymes, and other molecular targets. This high specificity not only enhances therapeutic efficacy but also reduces adverse side effects, leading to improved safety profiles in clinical applications. Their functional versatility allows them to act as anticancer, antimicrobial, antivirus, anti-immune, cytokine-inducing, hormonal, and neuromodulatory peptides, thereby expanding the range of treatable diseases.

Within this landscape, cytokine-inducing peptides, which elicit the secretion or modulation of specific cytokines from immune cells, represent a promising avenue for advanced immunomodulatory drug discovery or immune-modifying therapeutics by selectively engaging innate or adaptive immune pathways. These offer a level of control not easily achieved with small molecules or neutralizing antibodies. Interleukin-6 (IL-6)-inducing and interleukin-13 (IL-13)-inducing peptides exemplify complementary therapeutic peptides. They share common principles in cytokine regulation and act through receptor-mediated signaling pathways, influence downstream transcription factors, and modulate innate and adaptive immunity [4,5,6,7]. Since IL-6 is a pleiotropic mediator central to both innate and adaptive immunity, IL-6-inducing peptides predominantly elicit pro-inflammatory responses and are associated with acute-phase reactions, T-helper 17 (Th17) cell differentiation, and autoimmune and inflammatory conditions such as rheumatoid arthritis, IgA nephropathy, and the cytokine storm associated with severe COVID-19 [4,5,8,9]. In contrast, since IL-13 is a canonical T-helper 2 (Th-2) cytokine, IL-13-inducing peptides drive Th-2-mediated immune responses, contributing to allergic inflammation, parasitic infections, mucus production, and differentiation of naive human B cells toward IgG4- and IgE-producing cells [6,7]. Peptide-based vaccines and peptide kinoids targeting IL-13 demonstrated preclinical efficacy in attenuating airway inflammation and allergic phenotypes [10].

Despite this potential, experimental identification of these peptides is time-intensive and resource demanding. Thus, computational methods for their prediction are critically required to enable rapid and cost-effective screening of vast peptide sequence spaces [2,11,12]. To date, while machine learning (ML)- and deep learning (DL)-based methods have been proposed for identifying IL-6-inducing peptides [13,14,15,16,17,18] and for IL-13-inducing peptides [19,20,21], publicly available datasets for these cytokine classes remain extremely limited [15,19,20]. These methods implemented a broad range of ML and DL architectures in combination with diverse peptide encodings, including composition, position-order and physicochemical-based property representations, and language model embeddings such as Evolutionary Scale Modeling-2 (ESM-2) [22]. Kurata et al. [21] and Harun-Or-Roshid et al. [17] proposed an advanced ensemble learning framework in which a logistic regression model and genetic algorithms integrate the probability scores from over 100 single feature-based ML and DL models, respectively. Cao et al. developed a graph neural network model that incorporated three-dimensional structure information to identify IL-6-inducing peptides [23]. Despite these advances, predictive performance remains limited due to the severe scarcity of experimentally validated datasets [15,19,20].

To address data scarcity, conventional data augmentation techniques such as Synthetic Minority Oversampling Technique (SMOTE) and Kernel Density Estimation (KDE) were employed primarily in peptide prediction tasks [24,25,26,27,28]. To solve the limited dataset of anticancer peptides (ACPs), ACP-DA [24] and ACP-ADA [25] were proposed. ACP-DA encoded peptide sequences using a combination of binary profile features and AAindex-derived physicochemical properties and generated new samples in the feature space to enrich the training dataset [24]. ACP-ADA employed an AdaBoost-based framework that integrates binary profile features, amino acid indices, and amino acid composition [25].

On the other hand, generative AIs, including generative adversarial networks (GANs) [28,29], diffusion models (DMs) [30,31], and variational autoencoders (VAEs) [32], are expected as alternatives to conventional data augmentation methods. Such AI-based data augmentation would learn the underlying distribution of known functional peptides and generate diverse and informative representations of the sequence space while preserving key physicochemical and functional properties. Augmenting the training set with these high-quality generated or fake peptides would increase the prediction performance of classifier models. While early studies demonstrated the effectiveness of GAN-based augmentation for ACPs [28], the generative AI-driven data augmentation (GDA) strategies have hardly been established. It is required to propose some intelligible guidelines for GDA, including criteria for choosing model architectures of AIs, selecting high-quality generated peptides, and determining an appropriate augmentation ratio of the generated peptides to the training dataset.

To address the limitations arising from the small dataset sizes of IL-6- and IL-13-inducing peptides, we have developed a robust GDA framework that expands the peptide sequence space by incorporating AI-generated peptides into the training dataset. This development employed a case study-oriented proof-of-concept, in which we proposed the GDA strategy using a moderately sized anti-inflammatory peptide (AIP) dataset and subsequently applied the GDA framework to IL-6- and IL-13-inducing peptide predictors, named GDA-Preds.

## 2. Results

### 2.1. Development Strategy

**Figure 1:**
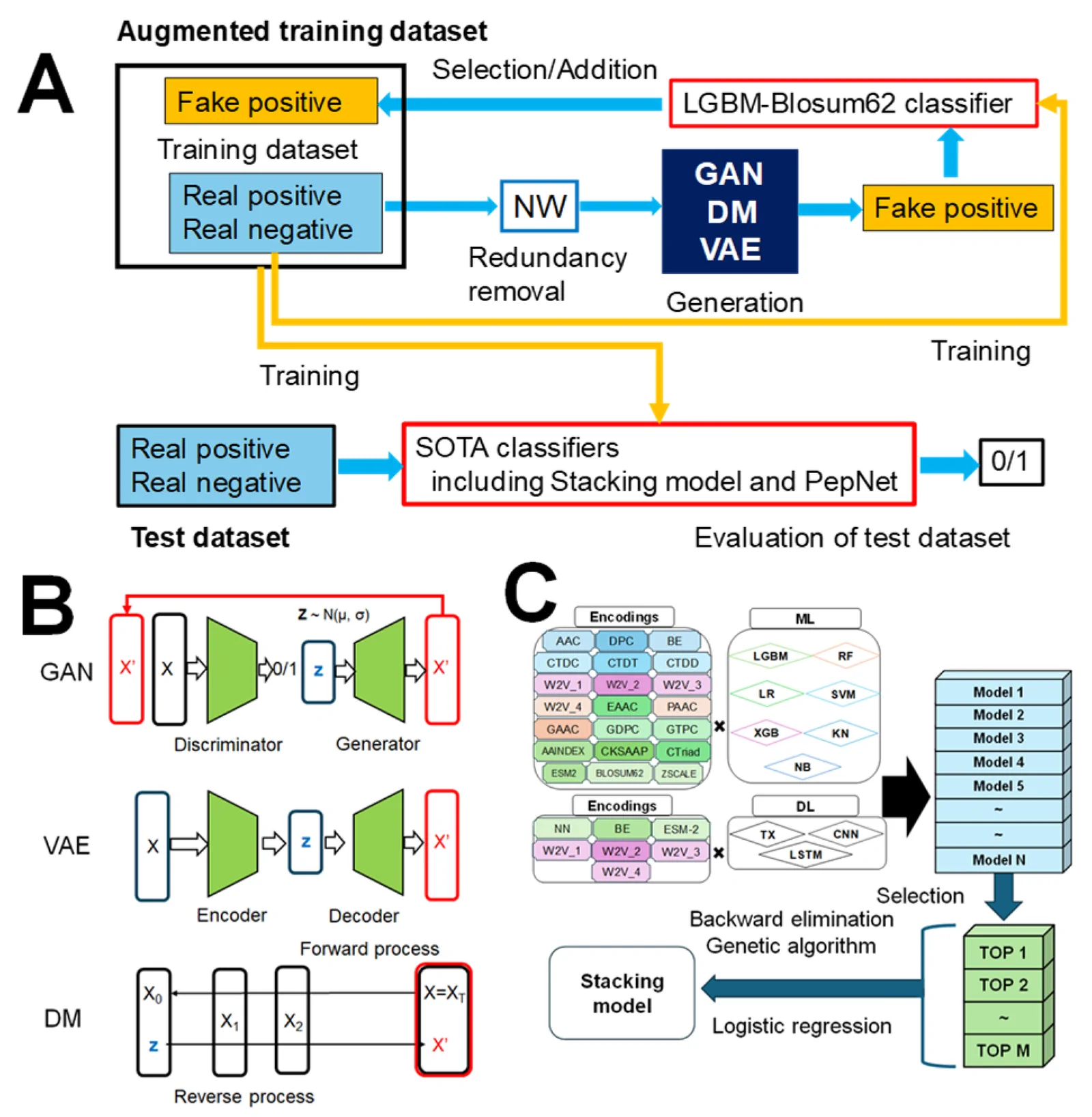
Development workflow of GDA-Pred. (**A**) GDA framework consisting of (1) generated fake peptide sequences from the real, positive peptides of the training dataset by generative AIs. (2) Select the generated peptides by a classifier constructed with the training dataset. (3) Add the generated peptides to the training dataset. (4) Construct classification predictors with the expanded training dataset. (5) Test the predictors with an independent test dataset. (**B**) Architectures of three generative AIs: GAN, DM, and VAE. (**C**) Our stacking classifiers for prediction of functional peptides.

Following a case study-oriented proof-of-concept approach, we proposed the GDA-Pred, as shown in Figure 1. The employed datasets are shown in Table 1. We first determined the best-performing setting for the four hyperparameters among the tested values using the relatively large AIP dataset and subsequently applied the reference setting to the IL-6- and IL-13-inducing peptide datasets. This method is adopted because the limited sizes of the IL-6 and IL-13 datasets make direct hyperparameter optimization difficult. The four key hyperparameters are: (i) the type of generative AI (GAN, DM, or VAE); (ii) the sequence identity cutoff value in the Needleman–Wunsch algorithm (NW) [33]-based redundancy removal (NW-RR); (iii) the probability threshold (PT) for selecting generated peptides via an Light Gradient Boosting Machine (LGBM) classifier; and (iv) the augmentation ratio (AR) that is defined as the ratio of selected peptides to the number of positive training peptides. The hyperparameters were determined using AIPs through a rigorous method with cluster-based 5-fold cross-validation (Figure S1).

### 2.2. Peptide Generation

**Figure 2:**
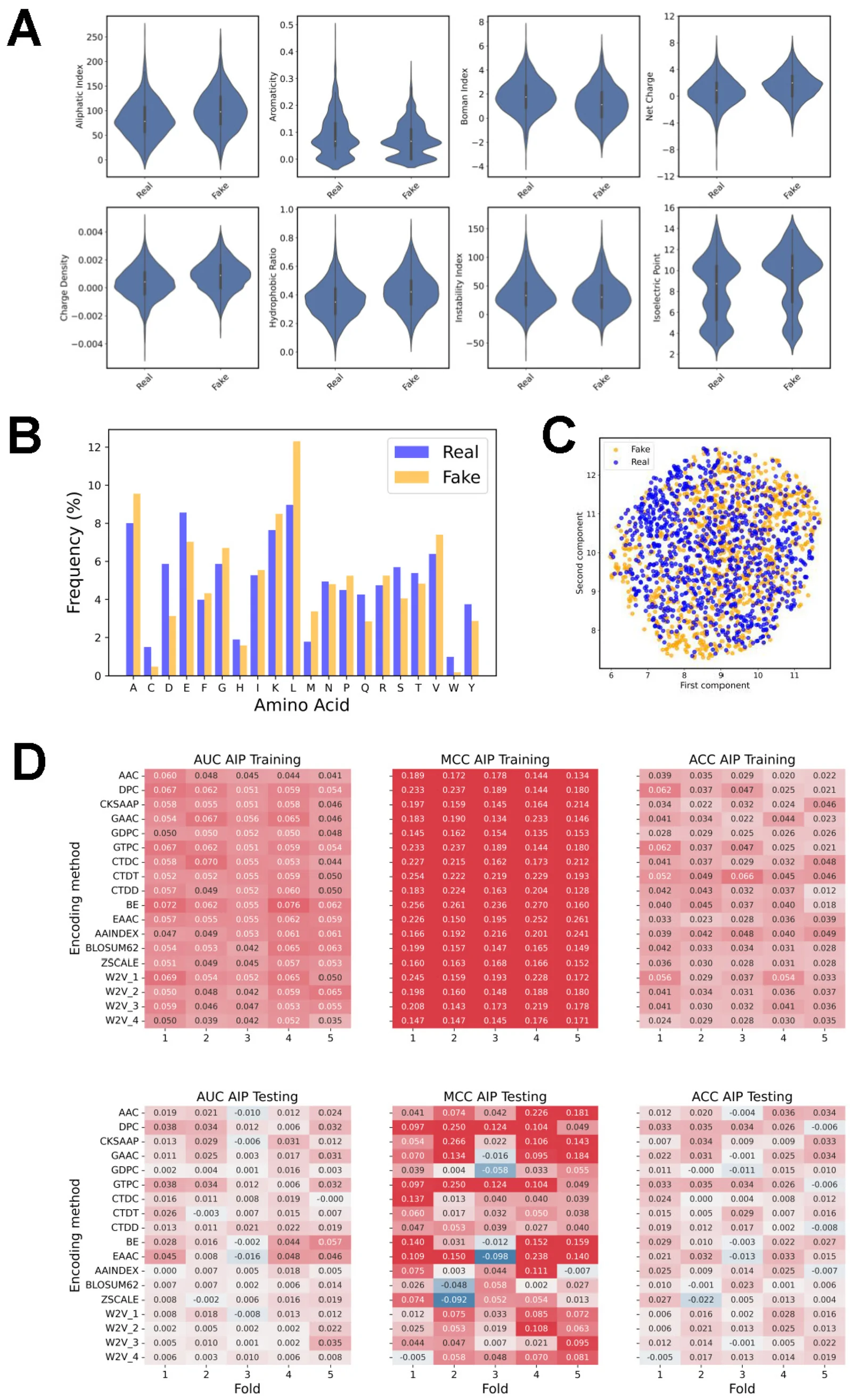
Quality assessment of GAN-generated AIPs and their impact on predictive performance. The GAN was trained on the AIP training dataset constructed using NW-RR with a sequence identity cutoff of 0.7. (**A**) Comparison of physicochemical property distributions between real and generated AIPs. (**B**) Frequency distributions of amino acid residues in real and generated peptide sequences. (**C**) UMAP visualization of encoding vectors representing real and fake AIPs. (**D**) PIRs of AUC, MCC, and ACC for 18 baseline classifiers trained on GAN-augmented AIP datasets, evaluated by stratified 5-fold cross-validation with cluster partitioning. Generated peptides were filtered using a LGBM classifier with BLOSUM62 encoding at PT of 0.5 and incorporated into the training dataset with an AR of 0.25. Red and blue colors indicate performance improvements and degradations, respectively.

The GAN model was trained using non-redundant, real positive sequences from the AIP training dataset, which were preprocessed with NW-RR using a similarity cutoff of 0.7. The GAN subsequently generated positive peptide sequences from Gaussian noise. The quality of the peptides generated was evaluated, as shown in Figure 2. The generated peptides exhibited slightly higher median values of aliphatic index, net charge, charge density, hydrophobic ratio and isoelectric point compared with the real peptides, whereas the remaining physicochemical properties were largely similar between the two groups (Figure 2A). Although the compositions of cysteine (C) and tryptophan (W) differed, those of the other amino acids were comparable (Figure 2B). The Uniform Manifold Approximation and Projection (UMAP) revealed that the embedding vectors derived from physicochemical property indices substantially overlapped between the distributions of the generated and real peptides (Figure 2C). These findings suggest that the generated sequences resemble the real positive sequences in both physicochemical characteristics and sequence patterns. The generated peptides via DMs and VAEs also exhibited similar properties to the real ones, as shown in Figures S2 and S3. In addition, the generated AIP peptides exhibited a novelty of 0.603 (Table 2), indicating that each generated peptide differed from its closest training peptide by approximately 60% on average. The generated peptide set showed a high diversity of 0.859 (Table 2), suggesting substantial sequence variation among the generated peptides.

### 2.3. Stratified 5-Fold CV with Cluster-Based Partitioning

The generated peptides were evaluated by an LGBM model with the BLOSUM62 encoding trained with the AIP training dataset to assign probability scores to them. We selected the generated peptides with probability values greater than 0.5 (PT) as augmentation data. To investigate the generalizability of the GDA, we added the selected AIP peptides to the training dataset with an AR of 0.25, trained the 18 baseline models with the expanded training dataset, and evaluated the models via stratified 5-fold CV with cluster-based partitioning (Figure S4). To highlight the effectiveness of GDA, the corresponding performance increase ratio (PIR) values of AUC, MCC, and ACC are illustrated in Figure 2D. In the training dataset, applying GDA enhanced the AUC, MCC, and ACC of all baseline models across every fold. Although the test dataset exhibited lower performance due to strict sequence similarity-based cluster partitioning, GDA improved AUC, MCC, and ACC in all but four of the 90 cases (18 encodings × 5 folds). Application of GDA distinctly increased the generalizability of AIP prediction.

### 2.4. Validation of GDA Hyperparameter Setting

In Figure 2D and Figure S4, we presented the GDA method with four reference hyperparameters: the type of GAN, a cutoff of 0.7, a PT of 0.5, and an AR of 0.25. Here, we validate these four hyperparameters. First, we investigated which generative AI was most suitable for GDA using a cutoff of 0.7, an AR of 0.25, and a PT of 0.5. Figure S5 shows the prediction performances of 18 baseline classifiers trained with the augmented dataset by GAN, DM and VAE via stratified 5-fold CV with cluster-based partitioning. In the training dataset, GAN and DM increased the PIR values of MCC for all the baseline classifiers across all 90 cases (18 classifiers × 5 folds), while VAE presented negative PIR values of MCC in several cases. In the test dataset, GAN increased the PIR value of MCC more than DM in many cases, while VAE showed lower PIRs of MCC than GAN and DM. Among the three generative models, GAN achieved the best performance, exhibiting slightly higher scores than DM and substantially higher than VAE.

Second, we analyzed how a cutoff of NW-RR influenced the performances of 18 baseline models trained with the GAN-expanded datasets. Using different cutoffs of 0.5, 0.6, 0.7, 0.8 and 0.9, we evaluated the AUC, MCC and ACC values of the 18 baseline classifiers with an AR of 0.25 and PT of 0.5 via stratified 5-fold CV with cluster-based partitioning, as shown in Figure S6. The AUC, MCC and ACC remained robust properties across a cutoff from 0.5 to 0.9 on both the training and test datasets with respect to baseline models. Based on these results, the cutoff value was set to 0.7 in this study.

Third, we investigated how a PT value altered the performances of 18 baseline models trained with the GAN-expanded datasets. Using different PT values of 0.0, 0.5 and 0.7, we evaluated the PIR values of MCC for the baseline classifiers with a cutoff of 0.7 and an AR of 0.25 via stratified 5-fold CV with cluster-based partitioning, as shown in Figure S7. In the training dataset, the PIR values of many baseline classifiers across all the folds increased with increasing PT, reaching saturation at a PT of 0.5. The GDA improved the MCC values for all baseline models. In contrast, in the test dataset, the number of the baseline classifiers exhibiting positive PIR values increased with PT across all folds, peaked at a PT of 0.5, and then declined at a PT of 0.7. At a PT of 0.7, overfitting was observed. Therefore, a PT of 0.5 was identified as the reference threshold for model generalization. These results indicate that a PT of 0.5 achieved an appropriate balance between sequence quality and diversity. In contrast, peptides generated with a PT of 0.0 exhibited excessive diversity, whereas those generated with a PT of 0.7 showed restricted diversity.

Fourth, we examined the effect of an AR on the prediction performance of 18 baseline models trained with the expanded datasets. Using AR values of 0.1, 0.25, and 0.4, we evaluated the PIR values of MCC for the baseline classifiers with a cutoff of 0.7 and a PT of 0.5 via stratified 5-fold CV with cluster-based partitioning, as shown in Figure S8. In the training dataset, the PIR values of the baseline classifiers across all folds increased progressively with the AR, attaining the highest values at an AR of 0.4. In contrast, in the test dataset, the number of the baseline classifiers exhibiting positive PIR values increased with AR across all folds, reached a peak at an AR of 0.25, and subsequently declined. At an AR of 0.4, overfitting was observed. Accordingly, the AR was determined to be 0.25.

Finally, we applied the GDA method to the benchmark datasets of AIPs. After removing redundancy from the benchmark training dataset with a NW-RR cutoff of 0.7, GAN was used to generate fake AIP sequences. The benchmark training dataset was augmented with an AR of 0.2 by using the selected, generated peptides with a PT of 0.5. We trained our stacking classifier (PredIL6) and PepNet with the expanded training dataset, and evaluated the models on the test dataset, as shown in Table 3. Applying GDA to both PredIL6 and PepNet increased many statistical metrics compared to the control method, whereas the conventional data augmentation method (KDE) reduced SEN, ACC, MCC, and AUC. The integration of GDA improved their performances with respective increases of 0.4% and 0.6% in AUC, 1.2% and 1.0% in MCC, and 0.3% and 0.4% in ACC for the stacking classifier and PepNet. PepNet exhibited higher scores of PRE, ACC, MCC, and AUC than the stacking model, probably because PepNet was specifically developed for the identification of AIPs [34].

### 2.5. Effect of Dataset Size on GDA

**Figure 3:**
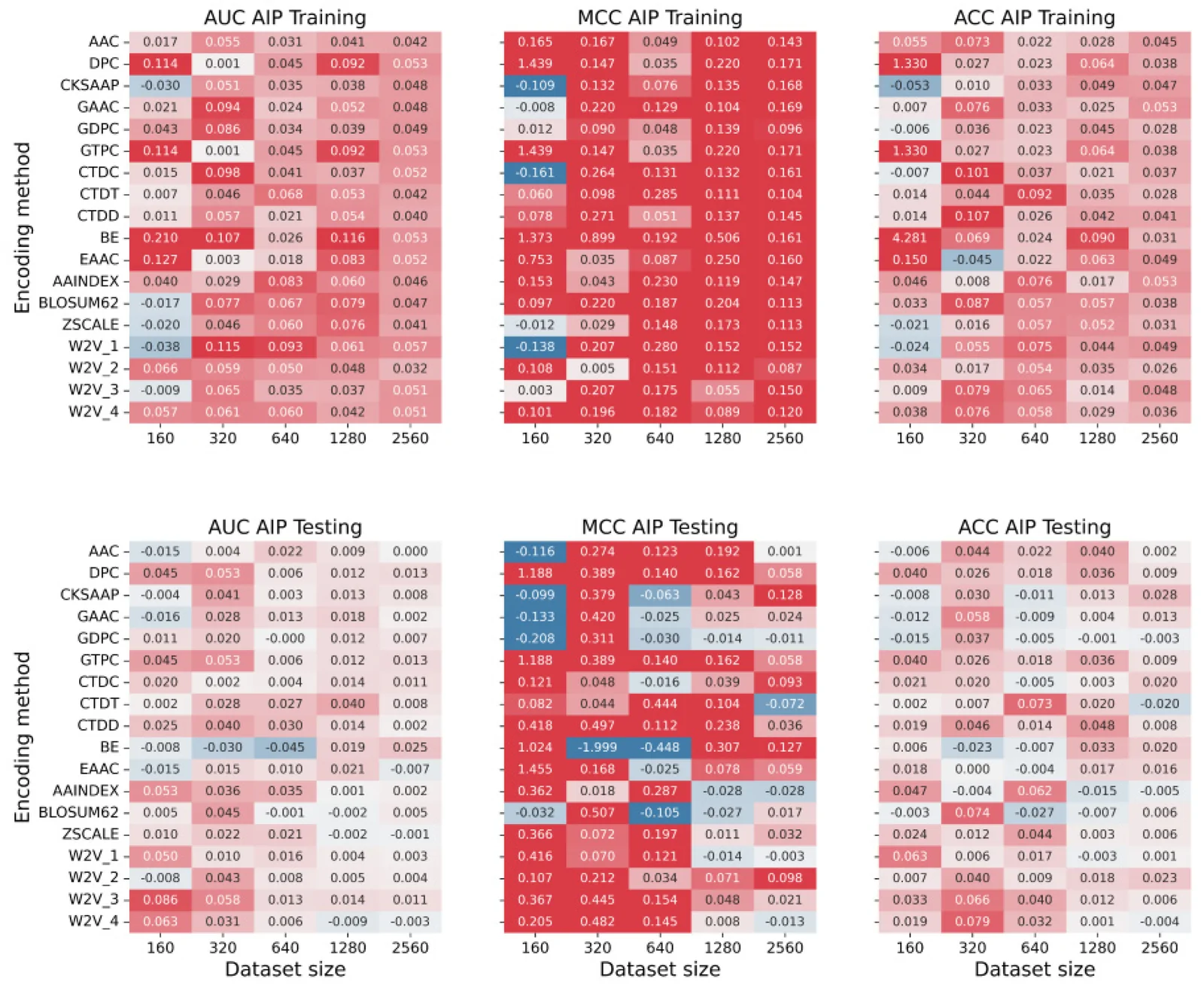
Effect of a training dataset size on PIRs of AUC, MCC, and ACC for 18 baseline classifiers trained by GAN-augmented AIP datasets. All classifiers were evaluated via 5-fold CV. The hyperparameter setting of GDA-GAN was a cutoff of 0.7, an AR of 0.25, and a PT of 0.5. Red and blue colors indicate performance improvements and degradations, respectively.

To examine how a training dataset size affects the effectiveness of GDA, the full benchmark dataset of AIPs was divided into training and test sets, while setting the ratio of the positive and negative data to be 1 and the five training datasets of different sizes were prepared. We then applied the GDA method to AIP datasets including 160, 320, 640, 1280, and 2560 samples, with an equal number of positive and negative samples, and evaluated the PIRs of AUC, MCC, and ACC on both the training and test sets, as shown in Figure 3. At a small dataset size of 160 (including 80 positive samples), some baseline classifiers exhibited negative PIR values in both the training and test datasets, whereas many other classifiers provided positive PIR values. At dataset sizes of 320 (including 160 positive samples) and 640 (including 320 positive samples), GDA enhanced the prediction performance of all baseline models on the training dataset and increased the number of the baseline models exhibiting positive PIR values in the test dataset. It indicates that GDA increases the generalizability. At larger dataset sizes of 1280 and 2560, GDA improved the prediction performance of all the baseline models in the training dataset; however, the PIR values of many classifiers decreased in the test dataset, indicating reduced generalizability.

We found that GDA was most beneficial when the dataset is moderate or limited, where newly generated data can fill gaps in the distribution. When the training dataset is small, GDA cannot capture the true data distribution. It easily overfits a few samples or covers only a narrow region of the sequence space. When the dataset is very large, the generative model learns a rich and comprehensive representation of the sequences; thus, additional generated data may not provide meaningful new information, which makes augmentation ineffective.

### 2.6. GDA Application to IL-6- and IL-13-Inducing Peptides

**Figure 4:**
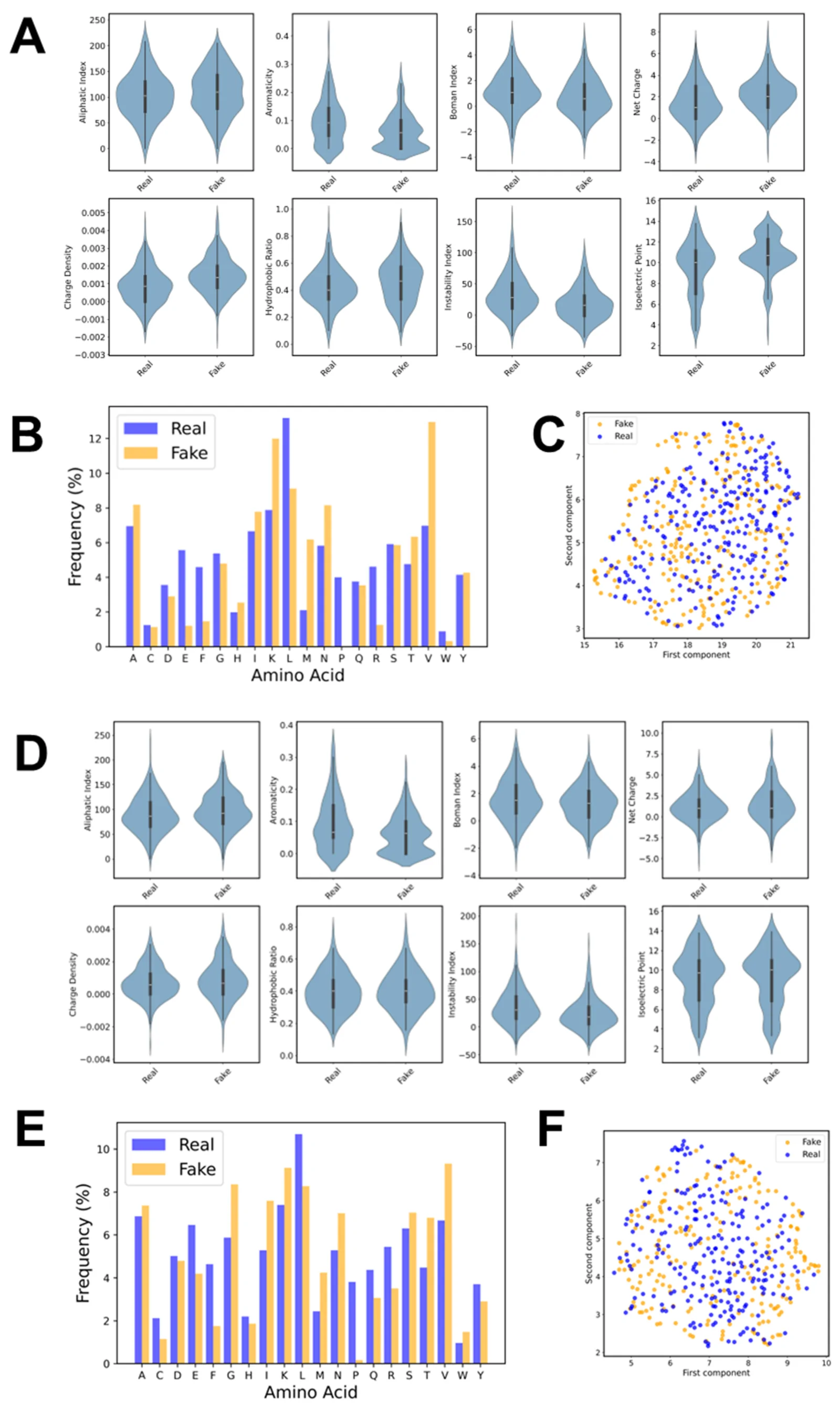
Quality assessment of the GAN-generated IL-6-inducing peptide sequences and IL-13-inducing peptide sequences. These two peptide sequences are generated from the training dataset built with NW-RR with a sequence identity cutoff of 0.7. (**A**–**C**) IL-6-inducing peptides: (**A**) Physicochemical properties. (**B**) Frequency distribution of amino acid residues. (**C**) UMAP distributions of the encoding vectors representing real and fake peptide sequences. (**D**–**F**) IL-13-inducing peptides: (**D**) Physicochemical properties. (**E**) Frequency distribution of amino acid residues. (**F**) UMAP distributions of the encoding vectors representing real and fake peptide sequences.

We applied the AIP-derived GDA to the identification of IL-6- and IL-13-inducing peptides. The qualities of the GAN-generated IL-6- and IL-13-inducing peptides derived from the benchmark training dataset were evaluated, as shown in Figure 4. For IL-6-inducing peptides (Figure 4A–C), the overall distributions of physicochemical properties between the generated and real peptides were broadly similar. However, noticeable differences were observed in the distribution shapes of aromaticity, hydrophobic ratio, and isoelectric point, as well as in the median values of aromaticity, Boman index, net charge, charge density, hydrophobic ratio, instability index, and isoelectric point. The amino acid composition analysis revealed that the frequencies of glutamic acid (E), phenylalanine (F), methionine (M), proline (P), arginine (R), and tryptophan (W) differed between the generated and real peptides, whereas those of the remaining residues were comparable. For IL-13-inducing peptides (Figure 4D–F), the distributions of most physicochemical properties between the generated and real peptides were similar except for aromaticity, and their median values were also comparable except for the instability index. The amino acid frequencies were largely consistent between the generated and real peptides, except for proline (P). Furthermore, UMAP visualization based on physicochemical property embeddings showed comparable distributions between the generated and real peptides for both IL-6 and IL-13 datasets. As shown in Table 4, the generated IL-6-inducing peptides achieved a novelty of 0.599 and diversity of 0.828, while the IL-13-inducing peptides showed a novelty of 0.608 and diversity of 0.849. The GAN-generated IL-6-/IL-13-inducing peptides exhibited moderately similar sequence and property patterns to the real peptide, whereas both peptide sets are substantially different from the training data and exhibit high sequence diversity.

**Figure 5:**
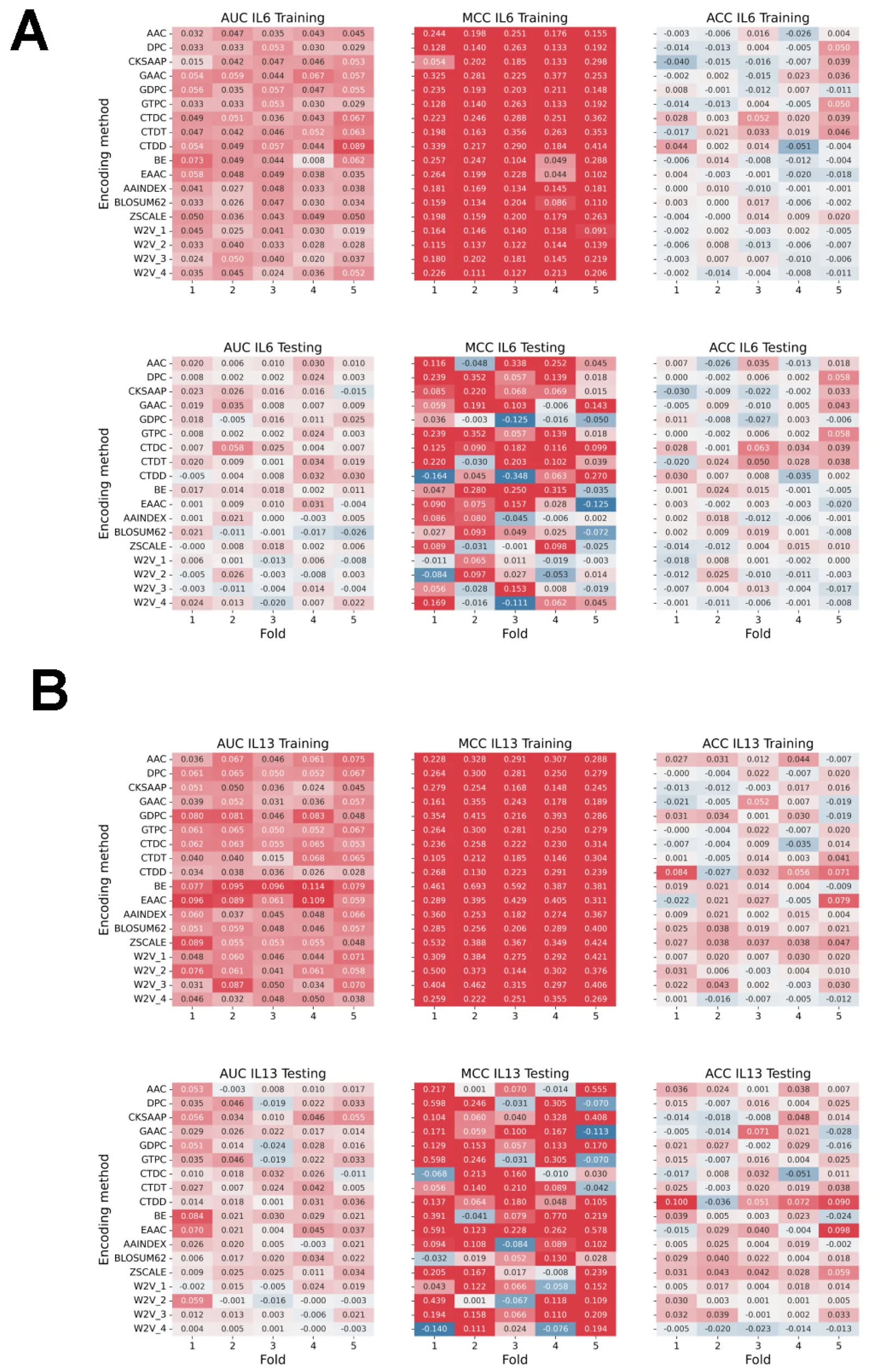
PIRs of AUC, MCC, and ACC for IL-6- and IL-13-inducing peptide prediction. (**A**) IL-6-inducing peptides. (**B**) IL-13-inducing peptides. Eighteen baseline classifiers were trained on datasets augmented with GAN-generated IL-6- or IL-13-inducing peptides and evaluated using stratified 5-fold cross-validation with cluster partitioning. The hyperparameter setting of GDA-GAN was a cutoff of 0.7, an AR of 0.25, and a PT of 0.5. Red and blue colors indicate performance improvements and degradations, respectively.

To demonstrate the effectiveness of GDA in identifying IL-6- and IL-13-inducing peptides, we trained the 18 baseline models with the GAN-augmented training dataset with a cutoff of 0.7, a PT of 0.5 and an AR of 0.25 and evaluated them via stratified 5-fold CV with cluster-based partitioning. Figure 5 illustrates the PIRs of AUC, MCC, and ACC on the training and validation datasets. Applying GDA enhanced the AUC, MCC, and ACC of all the baseline models across every fold on the training dataset for both cytokine-inducing peptides. The test dataset presented lower performance than the training one due to rigorous testing with cluster partitioning. In IL-6-inducing peptides, GDA improved AUC, MCC, and ACC in all but 27 of the 90 cases (18 encodings × 5 folds). In IL-13-inducing peptides, GDA improved AUC, MCC, and ACC in all but 16 of the 90 cases (18 encodings × 5 folds). The GDA substantially increased the generalizability of both cytokine-inducing peptides’ prediction in many cases.

To further evaluate the generalizability of the GDA method, it was incorporated into SOTA models (PredIL6, PredIL13, and PepNet), termed GDA-Preds, for the identification of IL-6- and IL-13-inducing peptides on their benchmark datasets, as shown in Table 5. For PredIL6, the incorporation of GDA resulted in improvements of 1.7% in AUC, 2.1% in MCC, and 0.2% in ACC, compared to the control methods, although the difference between the two methods was not statistically significant (DeLong test). Similarly, for PredIL13, GDA achieved increases of 1.0% in AUC, 24.3% in MCC, and 0.4% in ACC. GDA-Preds increased the performance of the PredIL6 and PredIL13 predictors for IL6- and IL13-inducing peptides, respectively. To confirm its effectiveness, GDA was applied to the PepNet predictor. The integration of GDA improved the performance for IL6- and IL13-inducing peptides, compared to the control methods, with respective increases of 1.3% and 1.0% in AUC, 5.2% and 14.0% in MCC, and 1.6% and 2.2% in ACC. Among the conventional data augmentation methods tested, KDE combined with PredIL6 achieved high SPE and PRE for IL-6-inducing peptides, whereas SMOTE and KDE combined with PePNet achieved high SEN and PRE, respectively, for IL-13-inducing peptides. Overall, GDA-Preds consistently achieved the best ACC, MCC, and AUC across the two cytokine-inducing peptides among the tested methods, surpassing the SOTA models in many metrics.

### 2.7. Robustness of Reference Hyperparameter Setting

We observed that the AIP-derived reference hyperparameter setting was also effective for the classification of two types of cytokine-inducing peptides. To evaluate the robustness and transferability of the reference setting, we systematically assessed the predictive performance of hyperparameter configurations in the vicinity of the reference setting across all three peptide datasets, as shown in Table S2. The reference setting yielded the best values for several metrics, including AUC, MCC and ACC, for AIPs. For IL-6- and IL-13-inducing peptides, it achieved the best AUC, although it did not consistently yield the best values for the other metrics. Overall, the reference setting demonstrated the best performance for AIPs and comparable performance for IL-6- and IL-13-inducing peptides.

## 3. Discussion

We have developed the GDA framework by determining the hyperparameters of GDA on a moderately sized dataset of AIPs and transferred the reference GDA to the IL-6 and IL-13 prediction tasks. The primary objective is not de novo discovery of novel peptides, but generation of reliable pseudo-positive samples that can be safely incorporated into the training dataset to improve the generalization performance of a downstream classifier.

Real functional peptides often have limited diversity or strong pattern bias and are only a tiny fraction of possible peptide space. Small peptide datasets may lead classifiers to overfit on a few sequences. Therefore, the GDA is required to produce high-confidence pseudo-positive sequences whose sequence patterns are not present in the original dataset, helping the classifier learn broader and weaker motifs than the original ones, while preserving physicochemical properties. Generated peptides are required to fill in gaps between real and fake sequences, smoothing the decision boundary and improving generalization to unseen peptides. In GANs, competition between a generator and a discriminator makes it possible to explore and design peptide sequence space. DMs learn the data distribution through a multi-step denoising process, gradually refining noise into a peptide sequence. This iterative refinement allows DMs to preserve detailed motifs and structural or physicochemical features. VAEs learn a structured, continuous latent space where similar peptides are located near each other, allowing interpolation between peptides and smooth exploration of sequence space. VAEs are likely to generate average-like or blurred samples. In this study, regardless of commonly noted advantages and disadvantages, GANs outperformed DMs and VAEs, suggesting that GANs generate realistic, diverse distributions of peptide sequences. Their adversarial training framework could force the generator to closely mimic the distribution of real data, while capturing diversity.

Since the prediction performance of the GDA-based baseline models remained robust as the NW-RR cutoff varied from 0.6 to 0.9, a cutoff of 0.7 was adopted for subsequent analyses. Removing sequence redundancy using NW is a crucial preprocessing step when training generative AI models for peptide design. Without any redundancy reduction, the model may simply memorize highly similar sequences rather than learning meaningful sequence–function relationships, resulting in overfitting. By filtering out duplicate and near-duplicate peptides, NW-RR enforces sequence diversity, compelling the model to capture generalizable patterns and enhancing its capacity to generate truly novel sequences.

A PT value of 0.5 was identified. PT serves as a critical hyperparameter for balancing sequence diversity and convergence: lower PT values promote the generation of more diverse peptide sequences, whereas higher PT values restrict sequence variability, potentially excluding peptides that are distant from the training distribution. Similarly, an AR of 0.25 was determined. The AR governs the extent of GDA applied to the training dataset. Although higher AR values improved model performance on the training set, they adversely affected test performance, suggesting that excessive augmentation can induce overfitting and diminish generalizability. AIP peptides and IL-6/IL-13-inducing peptides differ in their biological functions, sequence motifs, and physicochemical properties. We explicitly state that the PT and AR hyperparameters are not intended to capture biological characteristics of specific peptide classes. Instead, they are the hyperparameters that regulate the quality and quantity of generated peptides incorporated into the training dataset.

In terms of peptide quality, the GAN-generated peptides exhibited moderately similar sequences and property patterns to the real peptides. The moderate-to-high novelty score suggests that the generated peptides are not simple reproductions of the training data, while remaining sufficiently related to known peptide patterns. The high diversity indicates that the GDA explores a broad region of the peptide sequence space rather than generating highly similar variants, reducing the risk of mode collapse. Collectively, the GDA has strong potential for generating diverse and previously unseen peptide sequences that may serve as promising candidates for subsequent classification.

The size of the training dataset is a critical factor influencing the effectiveness of GDA. When the peptide dataset is too small, GDA is prone to overfitting due to the limited sequence diversity. Conversely, when the original dataset is sufficiently large and diverse, it encompasses most of the relevant sequence patterns. GDA provides minimal additional benefit, as the sequence space is effectively saturated.

The three generative AI models have a lot of tuning parameters (Table S1). GANs involve tuning parameters such as the deep network architecture, latent vector dimension, learning rate, and discriminator update frequency. DMs involve a noise schedule and number of diffusion steps, and sampling parameters. VAEs involve a latent dimension and KL divergence weights. Since optimization of these parameters was an extremely time-consuming task due to calculation complexity, we did not intensively optimize these parameters in this study. The optimization of generative AIs will be extensively explored in future work.

Our results support the robustness and transferability of the AIP-derived reference hyperparameter setting to the classification of IL-6- and IL-13-inducing peptides. However, the effectiveness of GDA and its optimal hyperparameter setting may depend on various dataset characteristics, including sequence motifs, physicochemical properties, and dataset size. Therefore, the mechanisms underlying the observed robustness and transferability of the reference setting across different peptide classes remain to be elucidated.

When sufficiently large cytokine-specific datasets become available, task-specific hyperparameter optimization or nested cross-validation will be necessary to determine whether further performance improvements can be achieved.

## 4. Materials and Methods

### 4.1. Development Flow

As illustrated in Figure 1A, the development of GDA-Pred comprises four main steps: (1) Generate fake peptide sequences from the real, positive peptides of the training dataset by generative AIs. (2) Select the generated peptides via a classifier built with the training dataset. (3) Add the generated peptides to the training dataset. (4) Construct classification predictors with the expanded training dataset and determine key GDA hyperparameters. (5) Evaluate the prediction performance of the GDA-based classifiers (GDA-Pred).

### 4.2. Benchmark Datasets

A curated dataset of experimentally validated bioactive peptide sequences was obtained from publicly available peptide databases [15,19,20,35,36], as shown in Table 1. Sequences were filtered to include only peptides with experimentally confirmed activity to exclude non-standard amino acids and limited to a specified length range (e.g., 5–50 residues).

### 4.3. Cluster-Based 5-Fold Partitioned Datasets

Redundant sequences can bias generative models to overfit some common patterns and cause data leakage. To eliminate redundant peptide sequences from the benchmark dataset, we employed the Needleman–Wunsch (NW) algorithm [33]-based redundancy removal method (NW-RR) using a sequence identity cutoff of 0.4, 0.5, 0.6, 0.7, 0.8, and 0.9. Generally, validation datasets that share sequence patterns with the training data can overestimate predictive performance. To avoid this problem, we adopted a 5-fold cross-validation (CV) with cluster-based partitioning (Figure S1). First, all-against-all global pairwise alignment scores between peptide sequences were computed using the NW algorithm implemented in Biopython’s pairwise2 module [37], with standard scoring parameters (match = +1, mismatch = −1, gap open = −10, gap extend = −0.5). The resulting similarity matrix was transformed into a dissimilarity matrix by subtracting each score from the maximum observed score. To reduce the high-dimensional similarity space, we applied classical multidimensional scaling (MDS) to project the sequences into a two-dimensional Euclidean space. Subsequently, k-means clustering (k = 5) was performed on the MDS coordinates to partition sequences into 5 groups. To ensure balanced dataset sizes, we randomly down-sampled each cluster to match the size of the smallest cluster.

### 4.4. Generation of Peptide Sequences by Generative AIs

Three generative AIs (GAN, DM, VAE) are used to generate fake, positive peptide sequences from the real, positive sequences of the non-redundant training dataset processed with NW-RR (Figure 1B). Peptide sequences are encoded by giving 6 physicochemical property indexes to each peptide residue. Output vectors are decoded into approximate residue vectors using cosine similarity. Details of model architecture, latent space and training parameters are shown in Table S1.

#### 4.4.1. GAN Architecture

Based on [29], we built a GAN consisting of a Two-Dimensional Convolutional Neural Network (2DCNN)-based generator and discriminator. These were trained in a minimax game framework with real positive sequences of functional peptides. The generator takes random noise vectors as input and generates fake peptide sequences. The discriminator receives the peptide sequences from both the real dataset and the generator to output a probabilistic score representing whether a given sequence originated from the true data distribution or is artificially generated.

#### 4.4.2. DM Architecture

Based on [30,31], we built a DM model to learn the generative distribution of functional peptides. The forward process gradually added Gaussian noise to the peptide embeddings over a predefined number of time steps. The reverse process was parameterized using a U-Net comprising CNN layers to predict and remove the noise at each step.

#### 4.4.3. VAE Architecture

To learn a compact latent representation of the peptide sequences, we implemented a sequence-to-sequence VAE model [32]. The encoder stacked multiple neural networks to encode the input peptide sequence into a fixed-length latent vector. The decoder, also based on Long Short-Term Memory (LSTM) layers, reconstructed the peptide sequence from the latent representation. Once the autoencoder is trained, novel peptide sequences are generated by sampling latent vectors from the learned latent space.

### 4.5. Conventional Data Augmentation Method

To generate positive sequences, we used two conventional data augmentation methods, Synthetic Minority Oversampling Technique (SMOTE) [26] and Kernel Density Estimation (KDE) [27], as control methods. SMOTE identifies its k-nearest neighbors in the feature space for each positive sequence. After a random neighbor is selected, a new sequence is generated along the line segment connecting the original sequence and its neighbor (k = 5). KDE is a non-parametric method for estimating the underlying probability distribution of a dataset. It estimates the data distribution by summing kernel functions, typically Gaussian kernels, centered at each data point. After fitting the KDE model with a bandwidth of 1.0 to the original data, new peptide sequences are generated by sampling from the estimated distribution.

### 4.6. Augmentation of Training Dataset

To assess the biological relevance of the generated peptides, we employed the Light Gradient Boosting Machine (LGBM) classifier with the BLOSUM62 encoding [38,39], trained with the real positive and negative sequences alone, to give a probability score to each generated peptide (Figure 1A). We selected the generated peptides with a probability of more than PT and then added them to the training dataset with an AR. This selection is not to assign labels to unlabeled real sequences but to select only high-confidence synthetic peptides. It is critically important to exclude irrelevant sequences or outliers.

### 4.7. Baseline Classifiers

We constructed 18 baseline models, the LGBM models combined with 18 different features, and evaluated the prediction performance of the models with the training and test datasets [17,21,35]. LGBM stands as a gradient boosting platform harnessing tree-based learning algorithms, presenting heightened efficiency and swiftness. The distinctive features of LGBM encompass adept handling of expansive datasets, superior effectiveness, and rapid execution. What distinguishes it is the employment of a histogram-based algorithm, discretizing continuous feature values into distinct bins, a departure from conventional tree-based methods.

The amino acid sequences were encoded using 18 different encoding techniques including Amino Acid Composition (AAC), Di-Peptide Composition (DPC) [40], Composition of k-spaced Amino Acid Pairs (CKSAAP) [41], Grouped Amino Acid Composition (GAAC) [42], Grouped Dipeptide Composition (GDPC), Grouped Tripeptide Composition (GTPC), Composition/Transition/Distribution (CTD) Composition (CTDC), CTD Transition (CTDT), CTD Distribution (CTDD) [43,44], Binary Encoding (BE), Enhanced Amino Acid Composition (EAAC) [45], Amino Acid indices (AAindex) [46], BLOSUM62 [47], and Word2Vec (W2V) with different Kmers of 1, 2, 3 and 4 [48]. All these sequence encoding techniques can be readily computed using the open-source software packages, including iLearn [45]. These encoding techniques capture a variety of properties, including compositional, position-order, evolutional and physicochemical properties, and linguistic patterns/distributions. Each of these encoding methodologies brings a unique lens to the sequence and contributes to a comprehensive and detailed analysis.

### 4.8. SOTA Classifiers

As SOTA models, we employed PredIL6 [17], PredIL13 [21], and PepNet [34]. In PredIL6 and PredIL13, seven ML algorithms (LGBM, eXtreme Gradient Boosting, random forest, support vector machine, logistic regression, naive Bayes, and k-nearest neighbor) and three DL architectures (convolutional neural network, attention, and LSTM) were integrated with a wide range of sequence encoding techniques, resulting in the construction of hundreds of baseline models (Figure 1C). The predicted probabilities from these baseline models were combined using a logistic regression- and genetic algorithm-based stacking approach [17,21]. The optimized stacking model was evaluated via 5-fold CV on the benchmark training dataset to ensure that the predicted probabilities were well calibrated with the true class labels. PepNet was developed as an interpretable neural network for predicting both AIPs and anti-microbial peptides (AMPs) by applying a pre-trained protein language model [34]. PepNet captures the peptide sequence information of residue arrangements and physicochemical properties using a residual dilated convolution block and then obtains function-related diverse information by introducing a residual transformer block that characterizes the residue representations generated by the pre-trained protein language model. The GDA-Preds, the SOTA classifiers trained with the GDA-expanded benchmark training dataset, were evaluated on the benchmark test dataset.

### 4.9. Quality of Generated Peptides

To examine the quality of generated (fake) peptides, we calculated the dipeptide frequency distribution and 8 physicochemical properties, including Aliphatic Index (Thermal Stability) [49], Aromaticity (Aromatic AA Content) [50], Boman Index (Molecular Compactness) [51], Net charge, Charge Density (Charge per Length) [50], Hydrophobic Ratio (Hydrophobicity) [52], Instability Index (Atmospheric Stability), and Isoelectric Point (Acid-Base Property) [53]. To visualize high-dimensional data of peptide sequence embeddings on a 2D map, Uniform Manifold Approximation and Projection (UMAP) is used as a dimensionality reduction technique [54]. In addition, two complementary metrics were highlighted: (i) novelty, defined as the minimum sequence distance between each generated peptide and the training peptides, and (ii) diversity, defined as the average pairwise sequence distance among generated peptides.

### 4.10. Metrics

Prediction performance was assessed using 6 established statistical metrics, each offering different insights into the prediction performance [55]. It includes accuracy (ACC), sensitivity (SEN), specificity (SPE), precision (PRE), Matthews correlation coefficient (MCC), and area under the receiver operating characteristic curve (AUC). Comprehensive descriptions and mathematical formulations of these metrics are available in previous studies. A threshold value that determines whether the probability scores are classified into positive and negative samples is adjusted so as to maximize MCC. To make clear the effectiveness of GDA, the performance increase ratio (PIR) is defined by:

(1) $$Performance\;Increase\;Ratio(PIR)=\frac{\mathrm{Performance}\;\mathrm{with}\;\mathrm{GDA}-\mathrm{Performance}\;\mathrm{without}\;\mathrm{any}\;\mathrm{GDA}}{\mathrm{Performance}\;\mathrm{without}\;\mathrm{any}\;\mathrm{GDA}}$$

where performance without any GDA is named control performance. A positive value of PIR indicates that GDA increases classification performance; a negative value indicates that GDA decreases it.

All programs were implemented in Python (3.11.7) using Biopython (1.83) [37], scikit-learn libraries (1.3.2) [56] and PyTorch (2.1.2) [57].

## 5. Conclusions

Based on a case study-oriented proof-of-concept, we proposed the GDA method using a moderately sized dataset of AIPs as an interpretable example. The AIP dataset provides sufficient data to optimize the key hyperparameters governing GDA performance: type of generative models, a sequence identity cutoff of NW-RR, a PT and an AR. Subsequently, the AIP-derived reference hyperparameter setting was applied to two independent testing problems: the identification of IL-6- and IL-13-inducing peptides, each characterized by limited dataset sizes. The application of GDA to the SOTA models increased the prediction performance of the two cytokine-inducing peptides, indicating the robustness and transferability of the reference hyperparameter setting across three peptides.

## Figures and Tables

**Table 1:** Benchmark datasets.

Peptide	Training Dataset		Test Dataset	
	Positive	Negative	Positive	Negative
IL6-inducing peptide	292	2393	73	598
IL13-inducing peptide	250	2326	63	582
AIP	1258	1887	420	629

**Table 2:** Quality of GAN-generated sequences.

Peptide	Novelty	Diversity
AIP	0.603	0.859

**Table 3:** GDA-enhanced prediction performances of SOTAs for identifying AIPs on the benchmark AIP test dataset. Two SOTA models were employed to predict AIPs with and without applying GDA. GDA is performed using GANs with a cutoff of 0.7, a PT of 0.5, and an AR of 0.25. For each hyperparameter setting, the upper and lower values represent the mean and standard deviation, respectively. Bold values indicate the best mean performance for each evaluation metric among the tested settings.

Peptide	Classifier	DA	SEN	SPE	PRE	ACC	MCC	AUC	*p*-Value
AIP	PredIL6	**GDA**	**0.627**	0.790	0.670	**0.725**	**0.424**	**0.766**	0.79
			0.056	0.050	0.035	0.009	0.013	0.001	
		SMOTE	0.394	0.837	0.625	0.660	0.263	0.696	NA
			0.157	0.082	0.048	0.024	0.068	0.018	
		KDE	0.334	**0.893**	**0.686**	0.669	0.281	0.724	NA
			0.174	0.069	0.040	0.030	0.079	0.011	
		**w/o**	0.624	0.789	0.665	0.723	0.419	0.763	Control
			0.048	0.036	0.021	0.002	0.004	0.004	
	PepNet	**GDA**	0.525	0.879	**0.745**	**0.737**	**0.441**	**0.779**	0.02
			0.049	0.022	0.024	0.013	0.029	0.033	
		SMOTE	0.386	**0.906**	0.733	0.698	0.351	0.739	NA
			0.183	0.104	0.062	0.039	0.093	0.069	
		KDE	0.537	0.832	0.686	0.714	0.392	0.752	NA
			0.079	0.055	0.068	0.042	0.090	0.067	
		**w/o**	**0.572**	0.843	0.711	0.735	0.437	0.775	Control
			0.055	0.039	0.054	0.032	0.069	0.068	

**Table 4:** Quality of GAN-generated peptides.

Peptide	Novelty	Diversity
IL-6-inducing peptide	0.599	0.828
IL-13-inducing peptide	0.608	0.849

**Table 5:** GDA-enhanced prediction performances of SOTAs for identifying IL6- and IL13-inducing peptides on the benchmark test dataset. Two SOTA models were employed to predict AIPs with and without applying GDA. GDA is performed using GANs with a cutoff of 0.7, a PT of 0.5, and an AR of 0.25. For each hyperparameter setting, the upper and lower values represent the mean and standard deviation, respectively. Bold values indicate the best mean performance for each evaluation metric among the tested settings.

Peptide	Classifier	DA	SEN	SPE	PRE	ACC	MCC	AUC	*p*-Value
IL6-inducing peptide	PredIL6	**GDA**	**0.458**	0.987	0.811	**0.929**	**0.575**	**0.882**	0.19
			0.054	0.004	0.042	0.003	0.028	0.005	
		SMOTE	0.332	0.991	0.855	0.919	0.490	0.868	NA
			0.030	0.003	0.029	0.003	0.024	0.007	
		KDE	0.405	**0.993**	**0.872**	0.929	0.565	0.868	NA
			0.056	0.003	0.039	0.006	0.047	0.008	
		**w/o**	0.438	0.987	0.813	0.928	0.563	0.867	Control
			0.042	0.005	0.043	0.001	0.014	0.009	
	PepNet	**GDA**	0.441	**0.951**	**0.532**	**0.896**	**0.425**	**0.831**	0.09
			0.087	0.017	0.058	0.011	0.054	0.011	
		SMOTE	**0.619**	0.863	0.367	0.837	0.387	0.798	NA
			0.142	0.052	0.088	0.035	0.057	0.054	
		KDE	0.575	0.897	0.433	0.863	0.419	0.806	NA
			0.123	0.061	0.103	0.043	0.048	0.039	
		**w/o**	0.458	0.933	0.506	0.881	0.404	0.820	Control
			0.101	0.035	0.170	0.020	0.027	0.016	
IL13-inducing peptide	PredIL13	**GDA**	**0.273**	0.987	0.729	**0.918**	**0.411**	**0.889**	0.56
			0.024	0.010	0.130	0.008	0.041	0.011	
		SMOTE	0.257	0.990	0.760	0.918	0.405	0.888	NA
			0.073	0.010	0.164	0.004	0.039	0.013	
		KDE	0.289	0.986	0.732	0.918	0.419	0.872	NA
			0.063	0.004	0.154	0.005	0.091	0.011	
		**w/o**	0.146	**0.997**	**0.852**	0.914	0.330	0.880	Control
			0.028	0.001	0.048	0.003	0.037	0.012	
	PepNet	**GDA**	0.321	**0.970**	0.552	**0.907**	**0.371**	**0.822**	0.41
			0.071	0.012	0.069	0.008	0.046	0.016	
		SMOTE	**0.464**	0.932	0.472	0.888	0.396	0.794	NA
			0.118	0.048	0.148	0.033	0.048	0.022	
		KDE	0.388	0.960	**0.611**	0.906	0.326	0.797	NA
			0.094	0.050	0.199	0.038	0.084	0.034	
		**w/o**	0.346	0.945	0.453	0.887	0.326	0.813	Control
			0.138	0.048	0.092	0.032	0.055	0.017	

## Data Availability

The programs of GDA and GDA-Pred and datasets are freely available at https://github.com/kuratahiroyuki/GDAPred (accessed on 3 December 2025).

## Supplementary Materials

The following supporting information can be downloaded at: https://www.mdpi.com/article/10.3390/ijms27177688/s1.

## Abbreviations

The following abbreviations are used in this manuscript:

GDA	Generative Data Augmentation
GDA-Pred	GDA-Prediction system
IL-6	Interleukin-6
IL-13	Interleukin-13
ACP	Anticancer Peptide
AIP	Anti-Inflammatory Peptide
Th-2	T-helper 2
Th17	T-helper 17
ML	Machine Learning
DL	Deep Learning
GAN	Generative Adversarial Network
DM	Diffusion Model
VAE	Variational AutoEncoder
SMOTE	Synthetic Minority Oversampling Technique
KDE	Kernel Density Estimation
LGBM	Light Gradient Boosting Machine
XGB	eXtreme Gradient Boosting
RF	Random Forest
SVM	Support Vector Machine
LR	Logistic Regression
NB	Naive Bayes
KNN	k-Nearest Neighbor
CNN	Convolutional Neural Network
LSTM	Long Short-Term Memory
KL	Kullback–Leibler
NW	Needleman–Wunsch
NW-RR	NW-based redundancy removal
PIR	Performance Increase Ratio
AR	Augmentation Ratio
PT	Probability Threshold
SEN	Sensitivity
SPE	Specificity
PRE	Precision
MCC	Matthews correlation coefficient
AUC	Area Under Curve
UMAP	Uniform Manifold Approximation and Projection
