# Supplementary material for: GDA-Pred: Generative AI-Driven Data Augmentation for Improved Prediction of IL-6 and IL-13-Inducing Peptides
Source: Int J Mol Sci. 2026 Aug 27;27(17):7688. doi: 10.3390/ijms27177688 (PMC13566239; doi:10.3390/ijms27177688)
Supplement: Supplementary file 1 [file ijms-27-07688-s001.zip › ijms-4488476-supplementary.pdf]

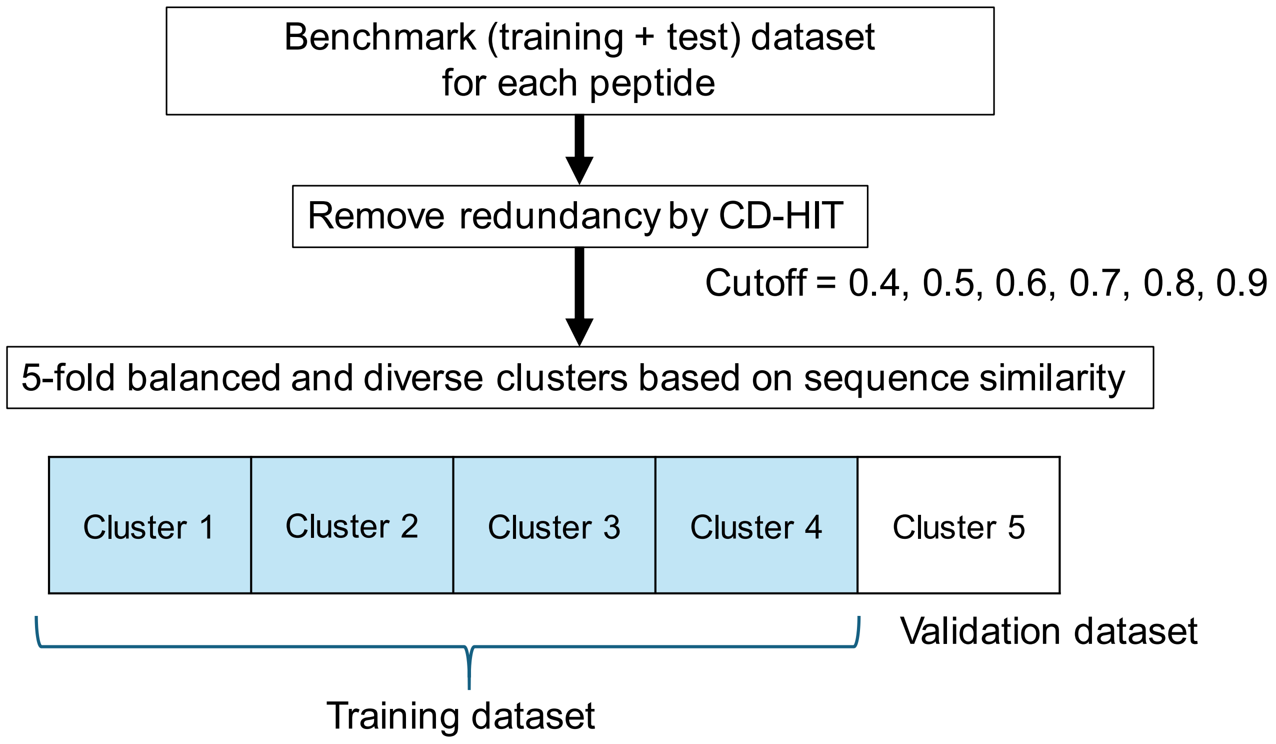

Figure S1 Stratified 5-fold CV with cluster-based partitioning for rigorously testing the generalizability of predictors.

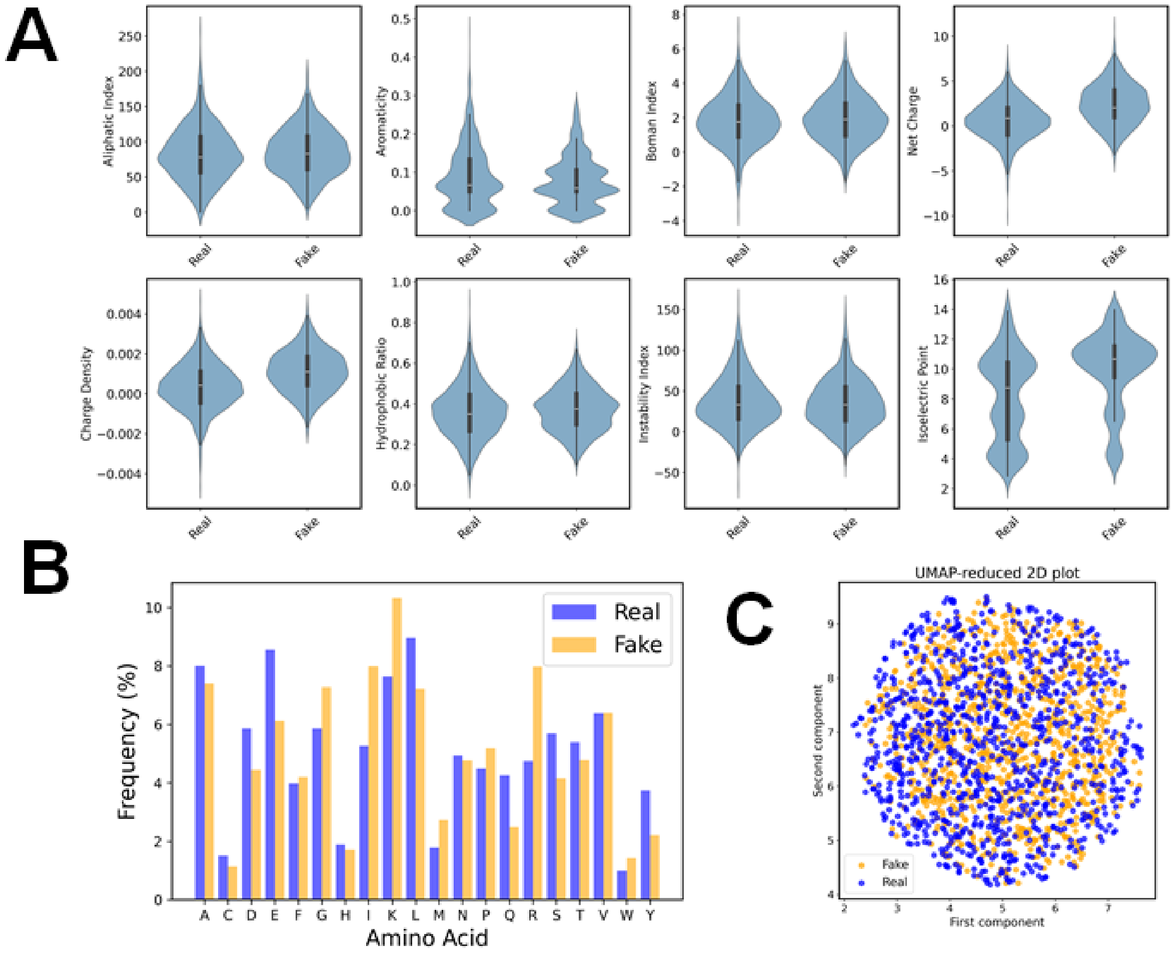

Quality of DM-generated sequences of AIPs

Peptide	Novelty	Diversity
AIP	0.614	0.875

Figure S2 Evaluation of the quality of AIP fake peptide sequences that DMs generate from the real, positive peptides of the training dataset. The fake peptides correspond to the generated peptides. The fake AIPs are generated from the non-redundant training dataset built with NW-RR with a sequence identity cutoff of 0.7. (A) Physicochemical properties. (B) Frequency distribution of amino acid residues. (C) UMAP distributions of the encoding vectors of real and fake peptide sequences.

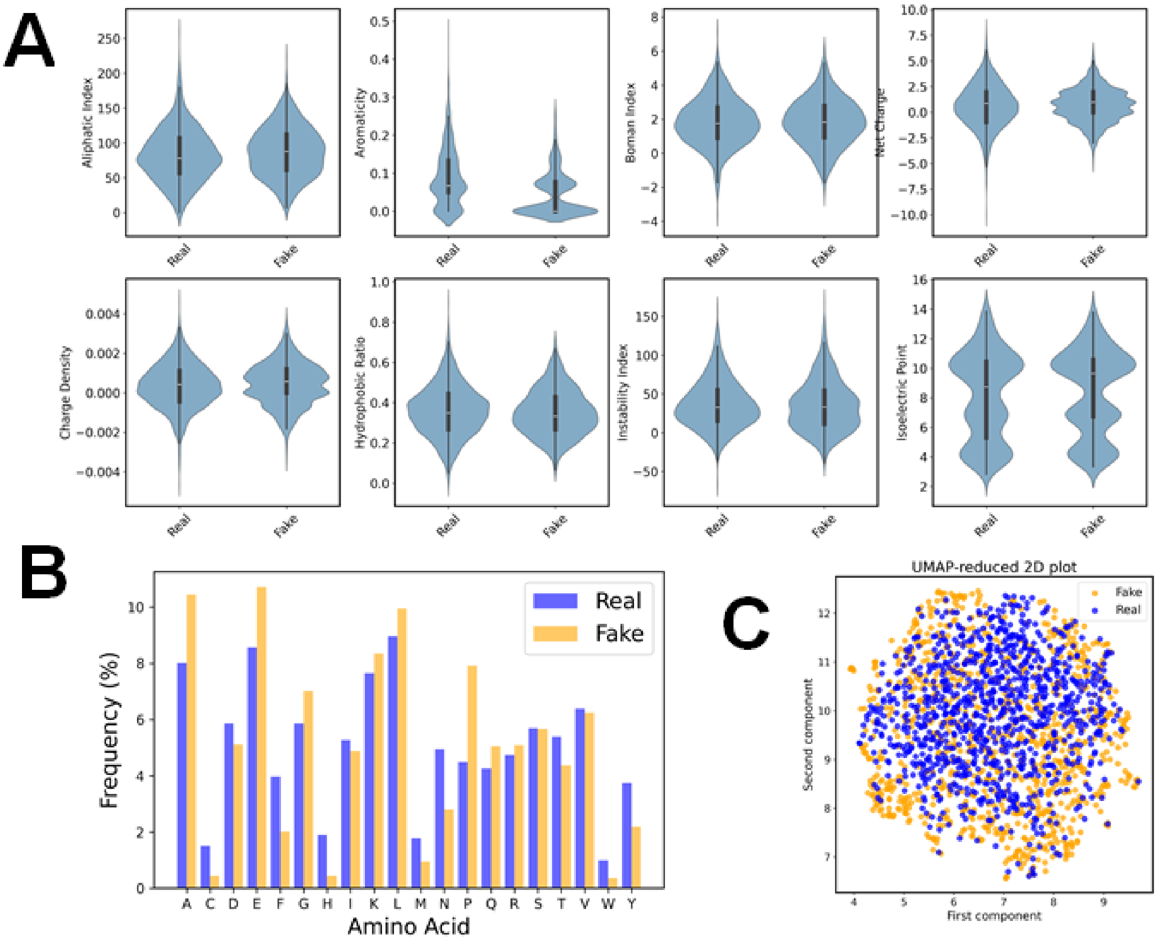

Quality of VAE-generated sequences of AIPs

Peptide	Novelty	Diversity
AIP	0.530	0.829

Figure S3 Evaluation of the quality of AIP fake peptide sequences that VAEs generate from the real, positive peptides of the training dataset. The fake peptides correspond to the generated peptides. The fake AIPs are generated from the non-redundant training dataset built with NW-RR with a sequence identity cutoff of 0.7. (A) Physicochemical properties. (B) Frequency distribution of amino acid residues. (C) UMAP distributions of the encoding vectors of real and fake peptide sequences.

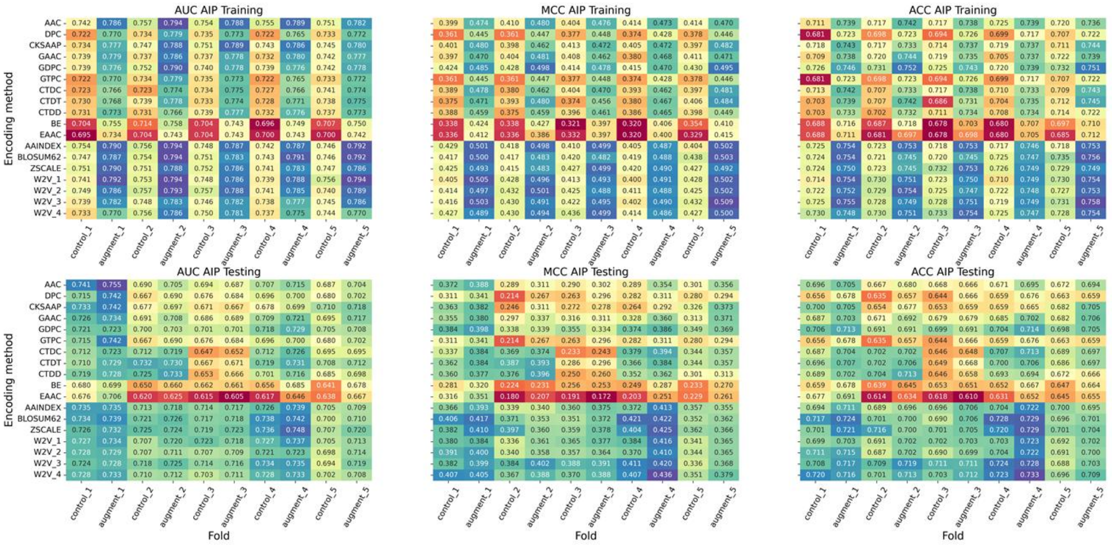

Figure S4 Prediction performance of 18 baseline classifiers trained by the augmented training dataset with GAN-generated AIPs via the stratified 5-fold CV with cluster partitioning. Fake AIP sequences were generated using GANs from a non-redundant training dataset built by CD-HIT with a sequence identity cutoff of 0.7. The generated peptides were filtered using an LGBM classifier with BLOSUM62 encoding at a PT of 0.5, and the selected sequences were added to the training dataset with an AR of 0.25.

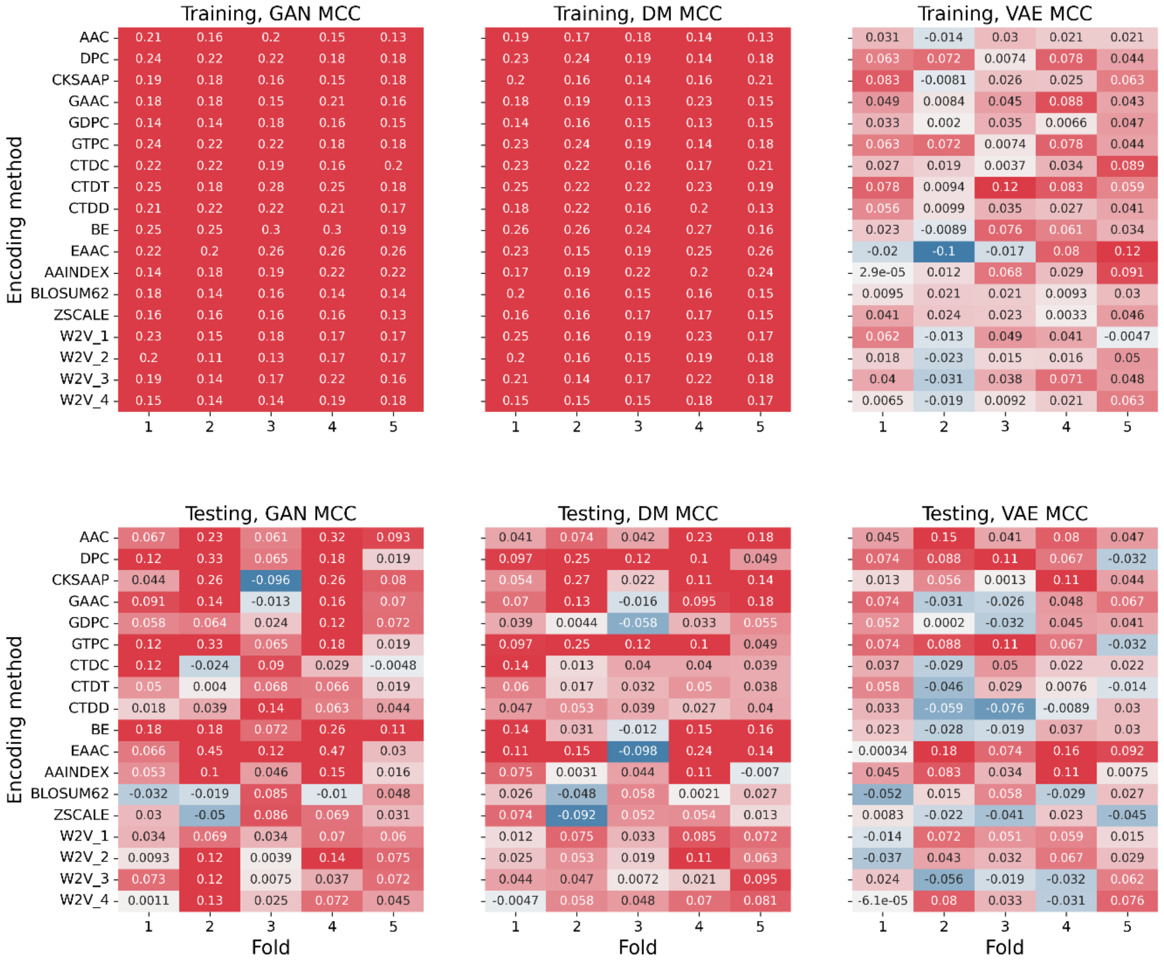

Figure S5 Comparison among the three generative AIs (GAN, DM, VAE) via stratified 5-fold CV with cluster-based partitioning, based on PIRs of MCC for 18 baseline classifiers trained by the augmented training dataset with three generative AIs-generated AIPs. Fake AIP sequences were generated using GANs from a non-redundant training dataset constructed by NW with a cutoff of 0.7. The generated peptides were selected at a PT of 0.5 using an LGBM classifier with the BLOSUM62 encoding and subsequently added to the training dataset with an AR of 0.25.

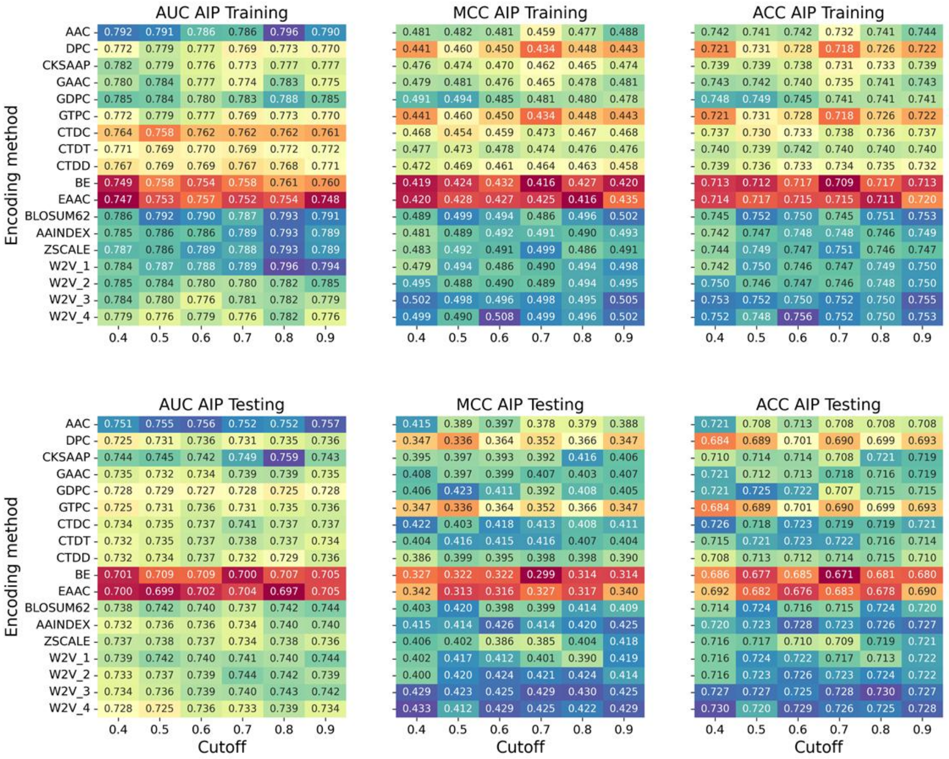

Figure S6 Effect of a sequence identity cutoff on AUC, MCC, and ACC for 18 baseline classifiers trained with the augmented training dataset with GAN-generated AIPs via the stratified 5-fold CV with cluster partitioning. Fake AIPs are generated from non-redundant training datasets built via NW with a cutoff of 0.5, 0.6, 0.7, 0.8 and 0.9. The generated peptides are selected at a PT of 0.5 by an LGBM classifier with BLOSUM62 encoding, and then added to the training dataset with an AR of 0.25.

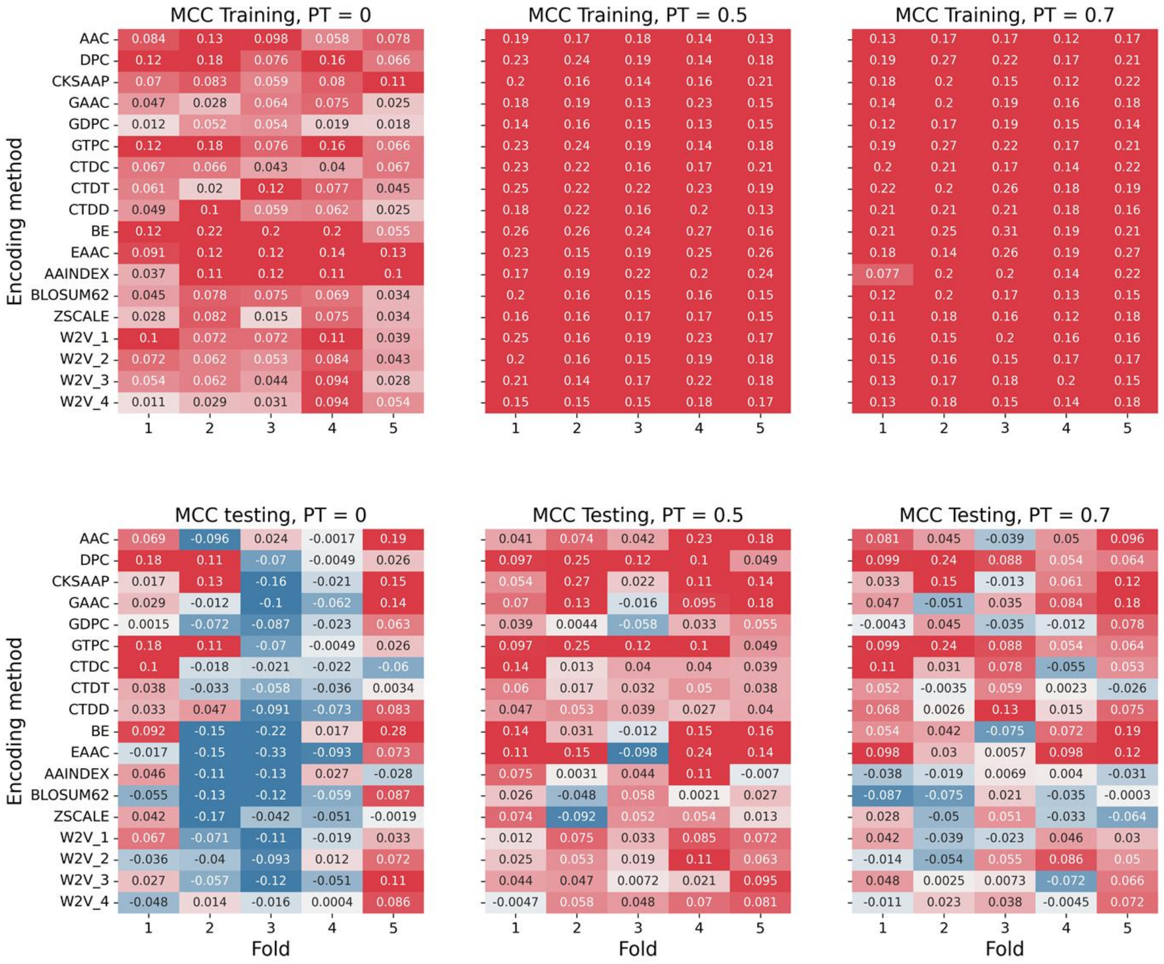

Figure S7 Effect of a PT on the PIRs of AUC, MCC, and ACC for 18 baseline classifiers trained by the augmented training dataset with GAN-generated AIPs via the stratified 5-fold CV with cluster partitioning. Fake AIP sequences were generated using GANs from a non-redundant training dataset constructed by NW with a cutoff of 0.7. The generated peptides were selected at PTs of 0, 0.5, and 0.7 using an LGBM classifier with the BLOSUM62 encoding and subsequently added to the training dataset with an AR of 0.25.

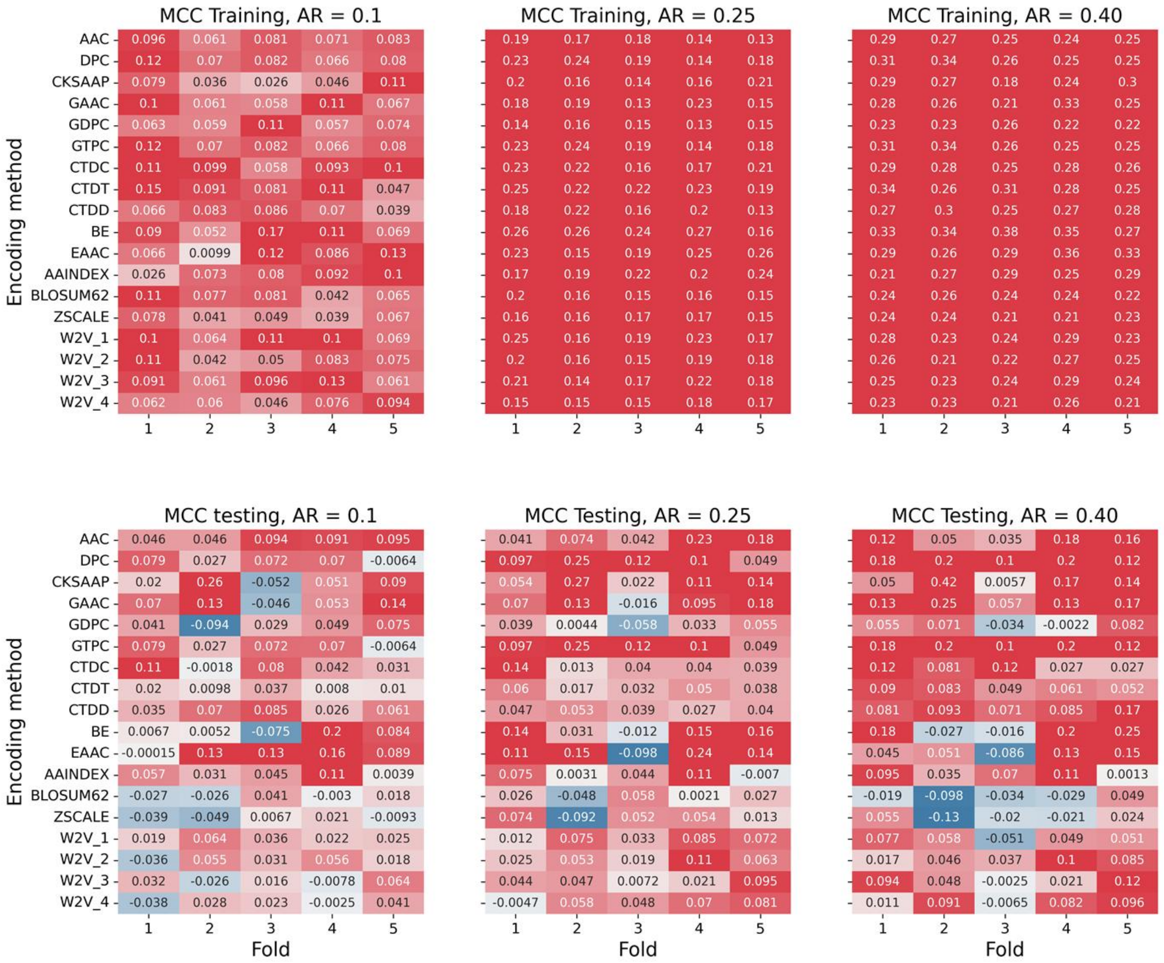

Figure S8 Effect of an AR on the PIRs of AUC, MCC, and ACC for 18 baseline classifiers trained by the augmented training dataset with GAN-generated AIPs via the stratified 5-fold CV with cluster partitioning. Fake AIP sequences were generated using GANs from a non-redundant training dataset constructed by NW with a cutoff of 0.7. The generated peptides were selected at a PT of 0.5 using an LGBM classifier with the BLOSUM62 encoding and subsequently added to the training dataset with ARs of 0.1, 0.25, and 0.4.

Table S1 Details of model architecture and tuning parameters for the three generative AIs of GANs, DMs, and VAEs.

GAN		
Module	Parameter	Value
Generator (G)	Number of layers	5
	Layer width	64
	Upsampling method	ConvTranspose2d
	Normalization	BatchNorm2d
	Activation function	ReLU
Discriminator (D)	Number of layers	4
	Downsampling method	Conv2d
	Activation function	ReakyReLU
Latent space	Distribution	Gaussian (Normal)
	Latent dimensionality	100
Training	Batch size	16
	Update ratio (number of D/number of G)	30
	Loss function	WGAN-GP (gradient penalty)
	Optimizer	Adam
	Learning rate of G	0.0001
	Learning rate of D	0.0001
	β1, β2 (Adam momentum terms)	0, 0.9
	Number of training epochs	1000
	Early stopping patience	20
	Stopping criteria (FID threshold)	generator_loss, dicriminator_loss
DM		
Module	Parameter	Value
U-Net model down sampling	Number of layers	2
	Downsampling method	Conv1d
	Downsampling activation function	ReLU
U-Net model middle block	Middle block method	Conv1d
	Middle block activation function	ReLU
U-Net model upsampling model	Number of layers	2
	Upsampling method	Conv1d
	Upsampling activation function	ReLU
Noise schedule (Diffusion Process)	Type of schedule	Linear
	Total number of diffusion steps	100
	β_min and β_max (start/end noise levels)	0.0001, 0.02
Latent space	Distribution	Gaussian
	Latent dimensionality	6
Training	Batch size	8
	Loss function	MSE
	Optimizer	Adam
	Learning rate	0.0001
	Number of training epochs	1000
	Early stopping patience	20
VAE		
Module	Parameter	Value
Encoder	Number of layer	2
	Method	Linear neural network
Decoder	Method	LSTM
		Linear neural network
Latent space	Distribution	Gaussian
	Latent dimensionality	32
Training	Batch size	128
	Loss function	Maximum Mean Discrepancy (MMD)
	Optimizer	Adam
	Learning rate	0.001
	Number of training epochs	10000
	Early stopping patience	20

Table S2 Performance of GDA-Pred for identifying three peptide classes on independent test datasets using hyperparameter settings in the vicinity of the AIP-derived reference setting. PePNet was used as the prediction model, and GAN-based data augmentation was performed with a sequence identity cutoff of 0.7. For each metric, the upper and lower values represent the mean and standard deviation, respectively. Bold values indicate the best mean performance for each evaluation metric.

Peptide	PT	AR	REC	SPE	PRE	ACC	MCC	AUC
AIP	0.4	0.1	0.567	0.810	0.685	0.713	0.399	0.752
			0.106	0.103	0.084	0.030	0.053	0.042
	0.4	0.4	0.527	0.835	0.691	0.712	0.388	0.742
			0.079	0.072	0.093	0.042	0.093	0.061
	0.5	0.25	0.525	**0.879**	**0.745**	**0.737**	**0.441**	**0.779**
			0.049	0.022	0.024	0.013	0.029	0.033
	0.6	0.1	**0.581**	0.800	0.687	0.712	0.404	0.761
			0.121	0.127	0.107	0.041	0.071	0.051
	0.6	0.4	0.548	0.837	0.693	0.721	0.406	0.760
			0.047	0.030	0.033	0.017	0.037	0.028
IL-6-inducing peptide	0.4	0.1	0.471	0.957	0.599	0.904	**0.475**	0.833
			0.055	0.025	0.121	0.016	0.037	0.019
	0.4	0.4	0.466	0.947	0.535	0.895	0.440	0.823
			0.022	0.021	0.097	0.018	0.056	0.016
	0.5	0.25	0.441	0.951	0.532	0.896	0.425	**0.831**
			0.087	0.017	0.058	0.011	0.054	0.011
	0.6	0.1	**0.564**	0.928	0.513	0.885	0.472	0.829
			0.090	0.016	0.062	0.018	0.077	0.019
	0.6	0.4	0.419	**0.964**	**0.661**	**0.904**	0.469	0.831
			0.073	0.042	0.168	0.030	0.059	0.007
IL-13-inducing peptide	0.4	0.1	0.292	0.959	0.471	0.894	0.303	0.797
			0.169	0.033	0.086	0.015	0.084	0.036
	0.4	0.4	0.311	0.953	0.479	0.890	0.322	0.759
			0.114	0.038	0.184	0.037	0.134	0.079
	0.5	0.25	0.321	**0.970**	0.552	0.907	0.371	**0.822**
			0.071	0.012	0.069	0.008	0.046	0.016
	0.6	0.1	**0.509**	0.953	0.577	0.906	**0.487**	0.812
			0.087	0.020	0.077	0.010	0.034	0.030
	0.6	0.4	0.356	0.967	**0.592**	**0.907**	0.404	0.789
			0.085	0.029	0.156	0.021	0.062	0.038
